# Altered regulation of flowering expands growth ranges and maximizes yields in major crops

**DOI:** 10.3389/fpls.2023.1094411

**Published:** 2023-01-19

**Authors:** Fan Wang, Shichen Li, Fanjiang Kong, Xiaoya Lin, Sijia Lu

**Affiliations:** Guangdong Key Laboratory of Plant Adaptation and Molecular Design, Guangzhou Key Laboratory of Crop Gene Editing, Innovative Center of Molecular Genetic and Evolution, School of Life Science, Guangzhou University, Guangzhou, China

**Keywords:** flowering, regional adaptation, crops, long day, short day

## Abstract

Flowering time influences reproductive success in plants and has a significant impact on yield in grain crops. Flowering time is regulated by a variety of environmental factors, with daylength often playing an important role. Crops can be categorized into different types according to their photoperiod requirements for flowering. For instance, long-day crops include wheat (*Triticum aestivum*), barley (*Hordeum vulgare*), and pea (*Pisum sativum*), while short-day crops include rice (*Oryza sativa*), soybean (*Glycine max*), and maize (*Zea mays*). Understanding the molecular regulation of flowering and genotypic variation therein is important for molecular breeding and crop improvement. This paper reviews the regulation of flowering in different crop species with a particular focus on how photoperiod-related genes facilitate adaptation to local environments.

Flowering is a central developmental process in the life cycle of plants. Plants must integrate internal factors and external cues to determine the optimal time to flower. This process is crucial for successful reproduction in all flowering plants but has added importance in crop species because of its major effect on yield. Daylength is one of the critical environmental cues that influence flowering time. Based on their flowering responses to daylength, plants can be categorized into three major types: long-day (LD) plants, short-day (SD) plants, and day-neutral (DN) plants. LD plants flower when the daylength is longer than a critical threshold, while SD plants flower when the daylength is shorter than a critical threshold. The flowering of DN plants is not affected by day length (Garner and Allard, 1920). LD plants, such as wheat (*Triticum aestivum*), barley (*Hordeum vulgare*), and pea (*Pisum sativum*), generally originated at higher latitudes, and tend to flower in late spring or early summer when periods of uninterrupted light extend past a certain threshold. SD plants, such as rice (*Oryza sativa*), soybean (*Glycine max*), and maize (*Zea mays*), originated at lower latitudes and tend to flower after they perceive a certain period of uninterrupted darkness (Brambilla et al., 2017). Here, we summarize the centers of domestication for major crop species and track their dissemination around the world. We also compare the different flowering regulation strategies in LD and SD crops, and discuss genotypic variation that arose during their dispersal.

## 1 Historical dissemination routes of major crops

### 1.1 LD crops

**Figure 1:**
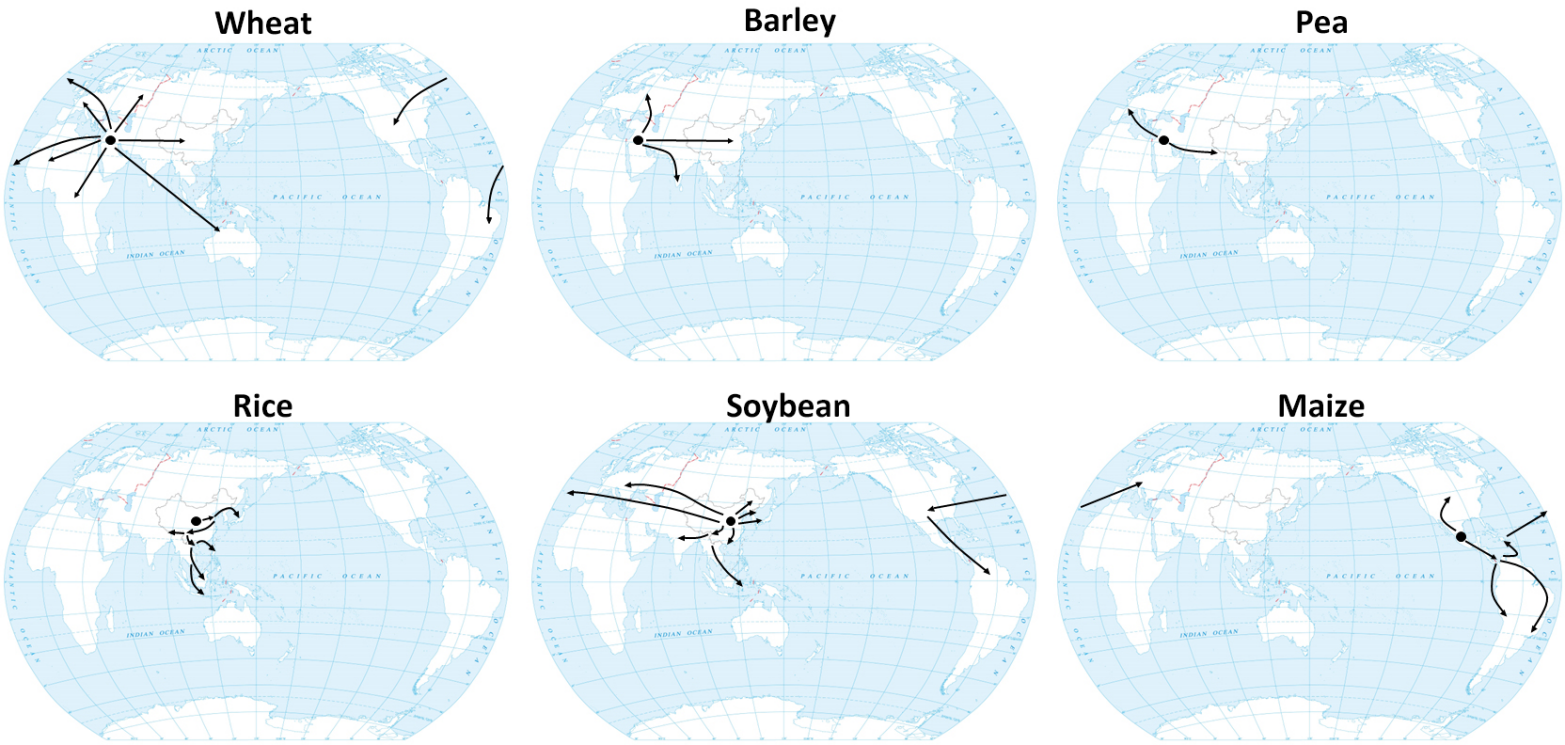
Domestication centers and hypothetical dissemination routes of major crops. Black arrows indicate the expansion scenario of wheat (Gohar et al., 2022), barley (Lister et al., 2018), pea (Jing et al., 2010), rice (Gutaker et al., 2020), soybean (Kihara, 1969; Hymowitz and Shurtleff, 2005; Stacey, 2008; Sedivy et al., 2017) and maize (Wang et al., 2021).

LD crops include wheat, barley, and pea, which were domesticated at a relatively high latitude in the Fertile Crescent, a narrow range that extends from 30°N to 40°N, and then were spread into different parts of world *via* different routes (**Figure 1**) (Zohary et al., 2012). The domestication of hexaploid bread wheat (*Triticum aestivum* L. ssp. *aestivum*) involved two hybridization events. The initial hybridization event took place between *Triticum urartu* (AA) and *Aegilops speltoides* (BB), resulting in emmer wheat, *Triticum durum* ssp. *dicoccum* (BBAA). The hexaploid *Triticum aestivum* ssp. *aestivum* (BBAADD) subsequently arose from a hybridization between domesticated emmer wheat (BBAA) and *Aegilops tauschii* (goat grass; DD) (Marcussen et al., 2014). Cultivated barley (*Hordeum vulgare* L. ssp. *vulgare*), however, was domesticated directly from a single wild progenitor, *Hordeum vulgare* ssp. *spontaneum* (Haas et al., 2019).

Pea (*Pisum sativum*) is also a LD crop. Its wild progenitor, *P. sativum* subsp. *elatius*, was initially domesticated in the Fertile Crescent as well. After domestication, *P. sativum* subsp. *elatius* began to be dispersed in two different directions. Eastward expansion into the Indian subcontinent and the Himalayan region gave rise to the Afghanistan germplasm group. The more prominent western expansion into Mediterranean Europe gave rise to the European domestic pea (*P. sativum* ssp. *sativum*) germplasm group, which eventually was developed into modern elite varieties (**Figure 1**) (Jing et al., 2010).

### 1.2 SD crops

SD crops include rice, soybean, and maize, which were domesticated at relatively low latitudes. There are two cultivated species of rice, Asian rice (*O. sativa* L.) and African rice (*O. glaberrima*). The archaeological record suggests that Asian rice was first domesticated in the middle and lower Yangtze River corridor in southern China as early as 9,000 years ago (**Figure 1**) (Doebley et al., 2006; Fuller et al., 2007; Wang et al., 2018). Two conflicting models exist to account for the domestication history of Asian rice: 1) the single domestication with introgression model and 2) the multiple independent domestications model. The first model suggests that wild rice can be divided into three major subpopulations: Or-I, Or-II, and Or-III. Population genetic analysis indicates that early-cultivated Asian rice, belonging to the *japonica* subspecies, was first domesticated from the Or-III-type *O. rufipogon* in southern China before spreading to other parts of Asia. Another subspecies of Asian rice, *indica*, was domesticated later by crossing *japonica* rice with local Or-I and Or-II-type wild rice, also belonging to *O. rufipogon* (Huang et al., 2012). By contrast, the multiple independent domestication model speculates that *japonica* was domesticated in China while *indica* was domesticated independently from local wild rice in China and India (Civáň et al., 2015).

Compared to Asian cultivated rice, African cultivated rice was grown in a limited area and, because of its low yield, has gradually been replaced by Asian rice (van Andel et al., 2016; Chen et al., 2019). African cultivated rice (*O. glaberrima*) was domesticated from its wild progenitor *O. barthii* around 3,000 years ago (Sweeney and McCouch, 2007; Wang et al., 2014; Huang et al., 2015). Some studies have proposed that African rice was domesticated in the Inner Niger Delta, while other studies suggest the domestication of African rice was multiregional (Huang et al., 2015; Snodgrass and Hufford, 2018; Choi et al., 2019; Veltman et al., 2019).

Soybean (*Glycine max*) was most likely domesticated from wild soybean (*Glycine soja*) in the Huang-Huai Valley of Central China (around 30–45°N) 5,000 years ago (Sedivy et al., 2017). Cultivated soybean then expanded to Korea, Japan, and other parts of Asia about 2,000 years ago (Kihara, 1969). In the 18^th^ century, soybean was disseminated to Europe and North America, and was introduced to Central and South America in the first half of the 20^th^ century (**Figure 1**) (Hymowitz and Shurtleff, 2005; Stacey, 2008).

Maize (*Z. mays* ssp. *mays*) was domesticated from its wild ancestor teosinte (*Z. mays* subsp. *parviglumis*) about 9,000 years ago in the Balsas region of southwest Mexico (Matsuoka et al., 2002; Piperno et al., 2009). From there, maize spread south and north to the rest of the Western Hemisphere around 1500 years ago. After Columbus arrived in the New World and brought maize to Europe, it rapidly spread around the world (**Figure 1**) (Brandolini and Brandolini, 2009; Nunn and Qian, 2010).

Through the broad dissemination of these major crops over their long history, the cultivation of both LD and SD crops has expanded from their original sites of domestication to the rest of the world. However, these species have a variety of distinct or partially overlapping strategies for regulating flowering time. Thus, a comparison of the molecular mechanisms underlying flowering time regulation is critical for identifying conserved and divergent mechanisms among these species.

## 2 Regulation of flowering time in LD plants

**Figure 2:**
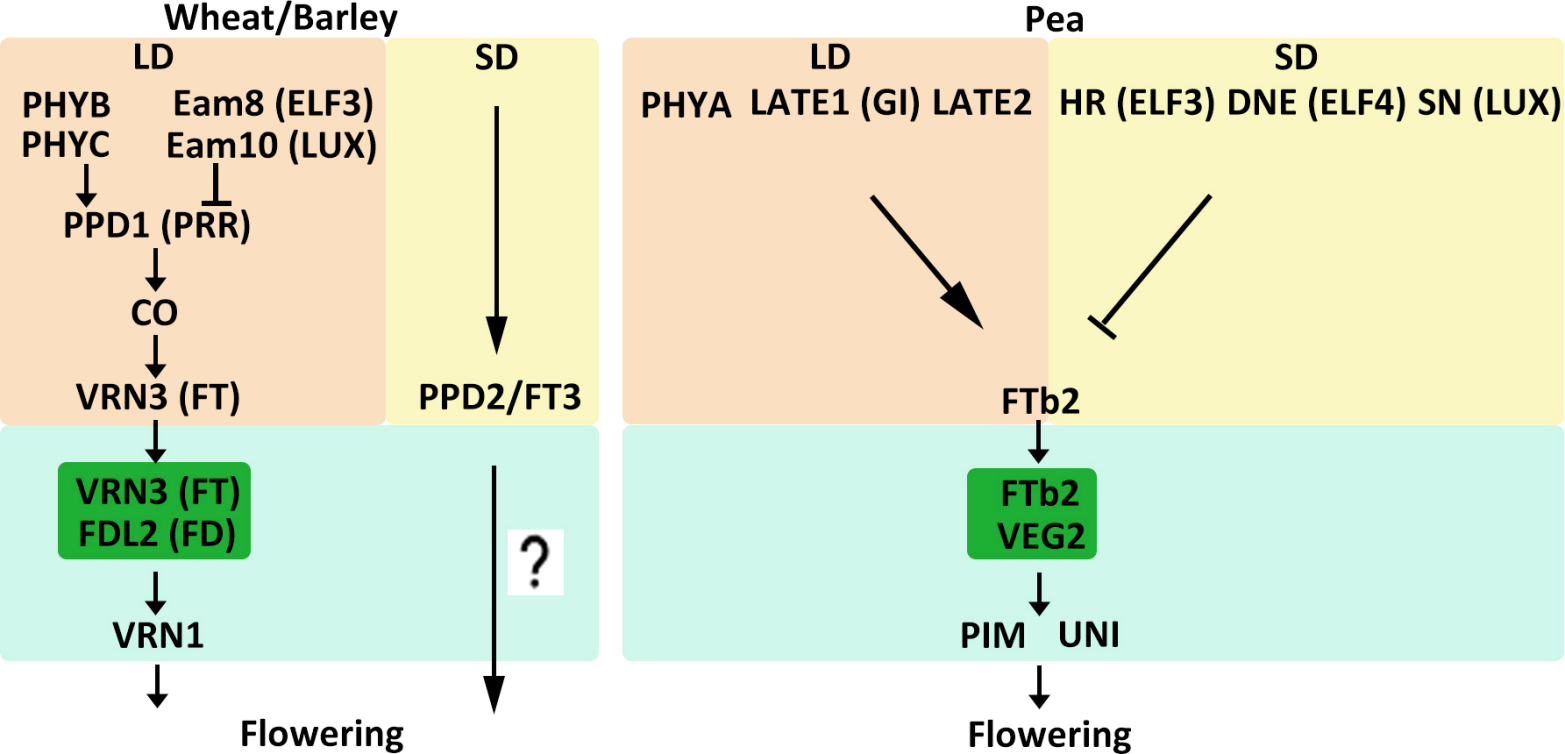
Model of flowering time control pathways in major LD crops. The different external and internal cues are highlighted in different colors. Positive and negative regulatory actions are indicated by arrows and lines with bares, respectively.

Temperate crops such as wheat, barley, and pea are LD plants, and as such their flowering is primarily controlled by daylength. For wheat and barley, flowering time under long day conditions is regulated by the *PHOTOPERIOD1* (*PPD1*) gene (**Table 1**, **Figure 2**) (Turner et al., 2005; Seki et al., 2011). *PPD1* encodes an ortholog of the Arabidopsis (*Arabidopsis thaliana*) PSEUDO-RESPONSE REGULATOR (PRR) protein, which is characterized by a pseudo-receiver and a CCT (CONSTANTS, CONSTANTS-like and TOC1) domain. *PPD1* expression is repressed by circadian clock genes EARLY FLOWERING 3 (ELF3) and LUX ARRHYTHMO (LUX) in wheat and barley (Faure et al., 2012; Mizuno et al., 2012; Zakhrabekova et al., 2012; Campoli et al., 2013; Alvarez et al., 2016). In addition, PHYTOCHROME family, PHYB and PHYC mediate activation of *PPD1* expression in the acceleration of wheat and barley flowering under LD conditions (Chen et al., 2014; Pankin et al., 2014; Pearce et al., 2016). LD conditions induce *PPD1* and upregulate *VERNALIZATION3* (*VRN3*), a homologue of the Arabidopsis gene *FLOWERING LOCUS T* (*FT*), by controlling *CONSTANS* activity to promote flowering (Turner et al., 2005; Chen et al., 2014). In Arabidopsis, FT protein moves from leaves to the shoot apical meristem (SAM) through the phloem (Turck et al., 2008). In the SAM, FT interacts with the bZIP transcription factor (TF) FLOWERING LOCUS D (FD) and binds to the promoters of *APETALA1* (*AP1*) and *FRUITFULL* (*FUL*) to induce the switch from vegetative to reproductive growth (Abe et al., 2005; Wigge et al., 2005). Similarly, in wheat, VRN3 interacts with an FD-like protein (TaFDL2) and subsequently binds to the promoter of *TaVRN1*, which is the ortholog of Arabidopsis *AP1* and *FUL* (Li and Dubcovsky, 2008). Under SD conditions, *VRN3* transcript levels are low (Yan et al., 2006). However, many varieties of wheat and barley can also flower under SD conditions, although flowering is delayed. Studies have identified a gene, *PPD2*, which is a paralog of *FT* referred to as *FT3*, that confers the ability to flower under SD conditions in barley and barley (Laurie et al., 1995; Faure et al., 2007; Kikuchi et al., 2009; Casao et al., 2011; Halliwell et al., 2016). However, how *PPD2* affects flowering under SD conditions and how *PPD2* regulates downstream genes remain unknown.

**Table 1:** Photoperiodic flowering genes of each crop.

Gene names	Arabidopsis gene	Target crops	Biological functions	References
*PHYB*	*AtPHYB*	Wheat	Phytoreceptor	(Pearce et al., 2016)
*PHYC*	*AtPHYC*	Wheat Barley	Phytoreceptor	(Chen et al., 2014; Pankin et al., 2014)
*PPD1*	*AtPRR7*	Wheat Barley	Circadian clock gene	(Turner et al., 2005; Seki et al., 2011)
*ELF3*	*AtELF3*	Wheat Barley	Circadian clock gene	(Faure et al., 2012; Zakhrabekova et al., 2012; Alvarez et al., 2016)
*LUX*	*AtLUX*	Wheat Barley	Circadian clock gene	(Mizuno et al., 2012; Campoli et al., 2013)
*CO*	*AtCO*	Wheat Barley	Transcription factor	(Griffiths et al., 2003)
*VRN3*	*AtFT*	Wheat Barley	Florigen	(Yan et al., 2006)
*PPD2*	*AtFT*	Wheat Barley		(Scarth and Law, 1983; Halliwell et al., 2016)
*FDL2*	*AtFD*	Wheat	Transcription factor	(Li and Dubcovsky, 2008)
*VRN1*	*AtAP1/AtFUL*	Wheat	Transcription factor	(Li and Dubcovsky, 2008)
*PHYA*	*AtPHYA*	Pea	Phytoreceptor	(Weller et al., 1997)
*LATE1*	*AtGI*	Pea		(Hecht et al., 2007)
*LATE2*		Pea		(Hecht et al., 2007)
*HR*	*AtELF3*	Pea	Circadian clock gene	(Weller et al., 2012)
*DNE*	*AtELF4*	Pea	Circadian clock gene	(Weller et al., 2012)
*SN*	*AtLUX*	Pea	Circadian clock gene	(Weller et al., 2012)
*FTa1*	*AtFT*	Pea		(Hecht et al., 2011)
*FTa2*	*AtFT*	Pea		(Hecht et al., 2011)
*FTb1*	*AtFT*	Pea		(Hecht et al., 2011)
*FTb2*	*AtFT*	Pea	Florigen	(Hecht et al., 2011)
*FTc*	*AtFT*	Pea		(Hecht et al., 2011)
*VEG2*	*AtFD*	Pea	Transcription factor	(Sussmilch et al., 2015)
*PIM*	*AtAP1*	Pea	Transcription factor	(Taylor et al., 2002)
*UNI*	*AtLFY*	Pea		(Hofer et al., 1997)
*PHYB*	*AtPHYB*	rice	Phytoreceptor	(Andrade et al., 2022)
*GI*	*AtGI*	rice		(Hayama et al., 2003)
*ELF3*	*AtELF3*	rice	Circadian clock gene	(Andrade et al., 2022)
*LUX*	*AtLUX*	rice	Circadian clock gene	(Andrade et al., 2022)
*PRR37*	*AtPRR3/7*	rice	Circadian clock gene	(Zhang et al., 2019)
*Ghd7*		rice	Transcription factor	(Xue et al., 2008)
*Hd1*	*AtCO*	rice		(Hayama et al., 2003)
*DTH8*	*AtHAP3B*	rice	Transcription factor	(Du et al., 2017)
*Hd6*		rice	Protein kinase	(Ogiso et al., 2010)
*Hd16*		rice	Protein kinase	(Hori et al., 2013)
*Ehd1*		rice	a B-type response regulator	(Zhao et al., 2015)
*RFT1*	*AtFT*	rice	Florigen	(Komiya et al., 2008)
*Hd3a*	*AtFT*	rice	Florigen	(Komiya et al., 2008)
14-3-3		rice	a phosphopeptide-binding protein	(Taoka et al., 2011)
*FD1*	*AtFD*	rice	Transcription factor	(Peng et al., 2007)
*MADS14*	*AtAP1/AtFUL*	rice	Transcription factor	(Yin et al., 2019)
*MADS15*	*AtAP1/AtFUL*	rice	Transcription factor	(Yin et al., 2019)
*MADS18*	*AtAP1/AtFUL*	rice	Transcription factor	(Yin et al., 2019)
*E3*	*AtPHYA*	soybean	Phytoreceptor	(Watanabe et al., 2009)
*E4*	*AtPHYA*	soybean	Phytoreceptor	(Liu et al., 2008)
*E2*	*AtGI*	soybean		(Watanabe et al., 2011)
*J*	*AtELF3*	soybean	Circadian clock gene	(Lu et al., 2017)
*LUX*	*AtLUX*	soybean	Circadian clock gene	(Bu et al., 2021)
*ELF4*	*AtELF4*	soybean	Circadian clock gene	(Lu et al., 2017)
*TOF11*	*AtPRR3*	soybean	Circadian clock gene	(Lu et al., 2020)
*TOF12*	*AtPRR3*	soybean	Circadian clock gene	(Lu et al., 2020)
*TOF16*	*AtLHY*	soybean	Circadian clock gene	(Dong et al., 2021)
*COL1a*	*AtCOL*	soybean		(Cao et al., 2015)
*COL1b*	*AtCOL*	soybean		(Cao et al., 2015)
*E1*		soybean	Transcription factor	(Xia et al., 2012)
*TOF5*	*AtFUL*	soybean	Transcription factor	(Dong et al., 2022)
*FT1a*	*AtFT*	soybean		(Liu et al., 2018)
*FT4*	*AtFT*	soybean		(Zhai et al., 2014)
*FT2a*	*AtFT*	soybean	Florigen	(Kong et al., 2010)
*FT5a*	*AtFT*	soybean	Florigen	(Kong et al., 2010)
*FDL19*	*AtFD*	soybean	Transcription factor	(Nan et al., 2014)
*SOC1*	*AtSOC1*	soybean	Transcription factor	(Nan et al., 2014)
*AP1*	*AtAP1*	soybean	Transcription factor	(Nan et al., 2014)
*LFY*	*AtLFY*	soybean	Transcription factor	(Nan et al., 2014)
*GI*	*AtGI*	maize		(Mendoza et al., 2012)
*COL3*	*AtCOL*	maize		(Jin et al., 2018)
*CONZ1*	*AtCO*	maize		(Miller et al., 2008)
*CCT*		maize	Transcription factor	(Huang et al., 2012; Huang et al., 2018)
*Ehd1*		maize	a B-type response regulator	(Zhong et al., 2021)
*ZCN8*	*AtFT*	maize	Florigen	(Zhong et al., 2021)
*MADS69*		maize	Transcription factor	(Liang et al., 2019)
*Rap2.7*		maize	Transcription factor	(Salvi et al., 2007)
*DLF1*	*AtFD*	maize	Transcription factor	(Muszynski et al., 2006)
*ZMM4*		maize	Transcription factor	(Danilevskaya et al., 2008)

Pea (*Pisum sativum*) is another crop grown in temperate environments. A number of loci related to flowering time have recently been identified in pea. Several of these are known to promote flowering in LD conditions. For instance, *phyA*, *late1*, and *late2* mutants all displayed a late-flowering phenotype under LD conditions (Weller et al., 1997; Hecht et al., 2007). *LATE2* has not yet been characterized, but *LATE1* is an ortholog of the Arabidopsis circadian clock-related gene *GIGANTEA* (*GI*) (Hecht et al., 2007). Loci that delay flowering under SD conditions have also been identified. Recessive alleles of *HIGH RESPONSE* (*HR*), *DIE NEUTRALIS* (*DNE*), and *STERILE NODES* (*SN*) can cause early flowering in SD conditions (**Table 1**, **Figure 2**) (Weller et al., 2009). *HR*, *DNE*, and *SN* have been identified as homologs of the circadian clock genes *ELF3*, *ELF4*, and *LUX*, respectively. (Weller et al., 2012; Liew et al., 2014). In Arabidopsis, ELF3, ELF4, and LUX work together to form the evening complex (EC) and participate in the evening loop of the circadian clock (Nagel and Kay, 2012). This mechanism might explain why *hr*, *dne*, and *sn* mutants have similar phenotypes. The legume *FT*-like genes are divided into three subclasses: *FTa*, *FTb*, and *FTc* (Surkova and Samsonova, 2022). Five *FT*-like genes have been identified in pea: *FTa1*, *FTa2*, *FTb1*, *FTb2*, and *FTc* (Hecht et al., 2011). *FTa* and *FTb* are expressed in leaves and are probably involved in vernalization and photoperiod responses, respectively. *FTc*, however, is expressed in the SAM and might be involved in the integration of signals from leaf-expressed *FT* genes (Hecht et al., 2011). *FTb2* is expressed in leaves, and grafting experiments suggest that it might generate a flowering stimulus that travels from leaves to the SAM and promotes flowering (Beveridge and Murfet, 1996; Hecht et al., 2011). FTb2 interacts with VEGETATIVE2 (VEG2), which is an ortholog of the Arabidopsis FD protein, and may participate in a florigen activation complex (FAC) to activate downstream floral meristem-identity genes (Sussmilch et al., 2015). Flowering regulation-related genes in pea also include *PROLIFERATING INFLORESCENCE MERISTEM* (*PIM*) and *UNIFOLIATA* (*UNI*), which are orthologs of Arabidopsis *AP1* and *LEAFY* (*LFY*), respectively (Hofer et al., 1997; Taylor et al., 2002).

## 3 Regulation of flowering time in SD plants

**Figure 3:**
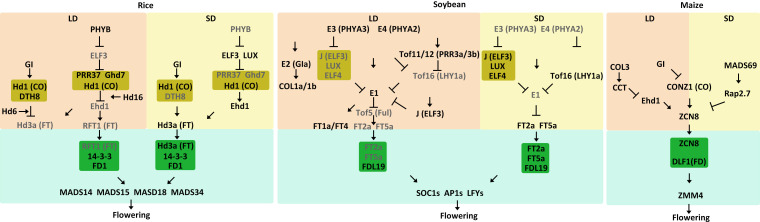
Model of flowering time control pathways in major SD crops. The different external and internal cues are highlighted in different colors. Positive and negative regulatory actions are indicated by arrows and lines with bares, respectively.

Rice is a SD crop, and its heading date is primarily determined by photoperiod sensitivity. Here, we summarize the current understanding of core molecular regulatory networks involved in rice flowering in both LD and SD conditions (**Table 1**, **Figure 3**). In LD conditions, rice has the GI-CO-FT pathway. *Heading date 1* (*Hd1*), an ortholog of the Arabidopsis *CO*, is expressed from nightfall to dawn. The diurnal expression of *Hd1* is regulated by *OsGI* (Hayama et al., 2003). *Heading date 3a* (*Hd3a*) is the ortholog of the Arabidopsis *FT* gene (Komiya et al., 2009). Different from Arabidopsis, rice *Hd1* negatively regulates *Hd3a* expression under LD conditions but positively regulate *Hd3a* expression under SD conditions (Hayama et al., 2003). The switch of Hd1 function is mediated by *DAYS TO HEADING 8* (*DTH8*) which encodes a CCAAT-box-binding TF. The DTH8-Hd1 complex increases H3K27 trimethylation at the *Hd3a* locus and represses *Hd3a* expression in LD conditions (Du et al., 2017). *Hd6* encodes one subunit of the protein kinase CASEIN KINASE 2 (CK2), which indirectly promotes Hd1-induced repression of *Hd3a* expression under LD conditions (Ogiso et al., 2010).

Aside from the conserved GI-CO-FT pathway, rice also has the *PHYB*-*ELF3*-*GHD7/OSPRR37*-*Ehd1*-*Hd3a*/*RFT1* regulatory pathway, which includes *Grain number, plant height, and heading date 7* (*Ghd7*), *Early heading date 1* (*Ehd1*) and *RICE FLOWERING LOCUS T1* (*RFT1*). Recently, it has been found that PHYB is activated under LD conditions and promotes the degradation of ELF3, thereby releasing the EC-mediated repression of *Ghd7* and *OsPRR37* (Andrade et al., 2022). *Ghd7*, which encodes a CCT-domain TF, is significantly induced by LD conditions (Xue et al., 2008). Ghd7 directly interacts with Hd1 to repress *Ehd1*, leading to suppression of the downstream gene *Hd3a* and *RFT1* to delay flowering (Xue et al., 2008; Weng et al., 2014). *Hd16*, which encodes CASEIN KINASE 1, enhances the repressive function of Ghd7 on *Ehd1* expression (Hori et al., 2013). Similarly, OsPRR37 and Hd1 together form a transcriptional repressor complex that downregulates *Ehd1* to suppress flowering under LD conditions (Goretti et al., 2017; Zhang et al., 2019).Both *RFT1* and *Hd3a* are orthologs of the Arabidopsis *FT* gene and function as florigens. However, RFT1 is a major LD activator whereas Hd3a is a major SD activator for rice. (Komiya et al., 2009). RFT1 protein, which is produced in leaves, moves to the SAM and forms a FAC with 14-3-3 and OsFD1, subsequently activating the expression of three *AP1/FUL*-like genes *OsMADS14*, *OsMADS15*, and *OsMADS18* (Peng et al., 2021).

Under SD conditions, ELF3 activity increases during periods of darkness, enabling the EC to reduce expression of key floral repressors such as OsPRR37 and Ghd7 in SD conditions. This converts Hd1 from a suppressor to an activator, promoting the expression of *Hd3a* at night (Andrade et al., 2022). DTH8 also interacts with Hd1, but Hd1 continues to act as an activator of *Hd3a* expression to promote flowering (Du et al., 2017). SD activator Hd3a protein is generated in leaves and moves to the SAM, where it interacts with 14-3-3 (Taoka et al., 2011). The Hd3a-14-3-3 complex enters the nucleus and forms an FAC with OsFD1, activating *OsMADS14*, *OsMADS15* and *OsMADS18* to promote floral transition (Komiya et al., 2008; Taoka et al., 2011; Tsuji et al., 2011; Yin et al., 2019).

Soybean is a typical SD plant that is very sensitive to photoperiod. Many important genes that control soybean flowering have been identified, including *E1*-*E11*, *J*, and quantitative trait loci including *Time of Flowering 5* (*Tof5)*, *Tof11*, *Tof12*, *Tof16*, and *Tof18* (Bernard, 1971; Buzzell, 1971; Buzzell and Voldeng, 1980; McBlain and Bernard, 1987; Ray et al., 1995; Bonato and Vello, 1999; Cober and Voldeng, 2001; Cober et al., 2010; Kong et al., 2014; Zhai et al., 2014; Wang et al., 2019; Lu et al., 2020; Dong et al., 2021; Dong et al., 2022; Kou et al., 2022). In the past decade, scientists have uncovered the molecular mechanisms of the key genes involved in soybean flowering regulation, namely the *E3/E4-E1-GmFTs* regulatory module (**Table 1**, **Figure 3**). *E3* and *E4* are the phytochrome genes *GmPHYA3* and *GmPHYA2*, respectively (Cober et al., 1996; Watanabe et al., 2009). E1 is a soybean-specific TF that is a core regulator of the flowering pathway (Xia et al., 2012). Under LD conditions, E3 and E4 function as photoreceptors that perceive light signals to influence downstream genes (Liu et al., 2008; Watanabe et al., 2009; Tsubokura et al., 2013). E3 and E4 physically interact with E1 to stabilize the E1 protein. In addition, E3 and E4 interact with LUXs and promote their degradation, releasing the suppression of EC on *E1* expression (Lin et al., 2022). E1 upregulates expression of the floral inhibitors *GmFT1a* and *GmFT4*, which further suppresses the expression of several downstream floral genes (Zhai et al., 2014; Liu et al., 2018). Although *GmFT1a* and *GmFT4* are highly expressed in leaves and are expressed in the SAM at low levels, there is no direct evidence to support GmFT1a and GmFT4 being transported from leaves to the SAM to inhibit flowering in soybean. (Zhai et al., 2014; Liu et al., 2018). Additionally, E1 represses transcription of *Tof5*, an ortholog of Arabidopsis *FUL*, by binding to its promoter. *E9* and *E10* encode GmFT2a and GmFT4, respectively, which are orthologs of Arabidopsis FT (Kong et al., 2014; Zhao et al., 2016; Samanfar et al., 2017). Tof5 physically associates with the promoters of the floral activators GmFT2a and GmFT5a to induce their expression (Kong et al., 2010; Dong et al., 2022). The downregulation of *GmFT2a* and *GmFT5a* ultimately represses the induction of *SUPPRESSOR OF OVEREXPRESSION OF CONSTANTS 1* (*GmSOC1*), *GmAP1*, and *GmLFY* (Nan et al., 2014). Thus, the *E3/E4-EC-E1-GmFTs* regulatory module delays flowering in LD conditions. In addition, Tof11 and Tof12, two PRR homologs, directly bind to the promoter of *LATE ELONGATED HYPOCOTYL* (*GmLHY*) to repress its expression. This prevents its transcriptional repression of *E1*, resulting in the *E3/E4-Tof11/Tof12-Tof16-E1-GmFTs* module (Li et al., 2020; Lu et al., 2020). Moreover, *E2* (*GIa*) encodes an ortholog of the Arabidopsis circadian clock component GI. The recessive *e2* allele leads to an early flowering phenotype by activating the expression of *GmFT2a* (Watanabe et al., 2011). Soybean has two additional *GI* orthologs, *GIb* and *GIc*, but their functions remain unknown (Wang et al., 2016). *CONSTANS-LIKE 1a* (*GmCOL1a*) and *GmCOL1b* are homologs of the Arabidopsis *CO*, and they suppress flowering in soybean under LD conditions (Cao et al., 2015; Cao et al., 2016). Compared to the conserved GI-CO-FT pathway in Arabidopsis, however, the functions of E2 and GmCOLs in soybean remain relatively unknown.

Under SD conditions, the functions of E3 and E4 become repressed (Xia et al., 2012). Functional reduction of E3 and E4 releases the suppression of Tof16, which encodes GmLHY1a, and J, which encodes an ortholog of the Arabidopsis EC component ELF3 (Lu et al., 2017; Dong et al., 2021). Tof16 and J control soybean flowering both additively and independently. Tof16 directly binds to the E1 promoter to suppress its expression (Dong et al., 2021). J combines with LUX and ELF4 to also inhibit the expression of E1 (Lu et al., 2017). Decreased expression of E1 releases the transcriptional suppression of GmFT2a and GmFT5a, further promoting flowering. In general, two regulatory modules are formed under SD conditions: EC-E1-GmFTs and Tof16-E1-GmFTs. Both GmFT2a and GmFT5a move from the leaves to the SAM and interact with the soybean ortholog of Arabidopsis FD, GmFDL19, to upregulate several downstream genes that promote flowering, like GmSOC1, GmAP1, and GmLFY (Nan et al., 2014).

Maize (*Zea mays* ssp. *mays*) was domesticated from its wild progenitor teosinte (*Zea mays* ssp. *parviglumis*) (Doebley et al., 2006). Modern maize and teosinte are, however, quite different from each other. Teosinte grows in tropical regions and requires SD photoperiods to induce flowering, while maize is grown at higher latitudes and is primarily photoperiod insensitive, with some varieties being DN plants (Minow et al., 2018). When early Native American farmers migrated to higher latitudes, they selected maize lines that were less dependent on SD photoperiods to flower. A CCT domain-containing gene *ZmCOL3* has been identified as an inhibitor of flowering, itself being inhibited in SD conditions but activated in LD conditions. In LD conditions, *ZmCOL3* becomes activated and directly induces *ZmCCT* transcription (**Table 1**, **Figure 3**) (Ducrocq et al., 2009; Hung et al., 2012; Yang et al., 2013; Huang et al., 2018). The ZmCCT protein binds to the promoter of *ZmEhd1*, which is homologous to the rice *OsEhd1* gene, repressing its transcription. Downregulation of *ZmEhd1* reduces expression of the florigen gene *ZEA CENTRORADIALIS 8* (*ZmZCN8*) (Zhong et al., 2021). Thus, this *ZmCOL3*-*ZmCCT*-*ZmEhd1*-*ZmZCN8* module regulates flowering in LD conditions. The *gi* mutation in maize, however, leads to an early flowering phenotype in LD conditions. Maize contains two homologs of the Arabidopsis *GI*, *ZmGI1* and *ZmGI2* (Mendoza et al., 2012). Transcription analysis has demonstrated that *ZmGI1* represses expression of *ZmZCN8* and *ZmCONZ1*, which is the homolog of Arabidopsis *CO* gene (Bendix et al., 2013). Although there is no evidence that *ZmCONZ1* activates *ZmZCN8* expression, the data suggest that *ZmCONZ1* is downstream of *GI1* but possibly upstream of *ZmZCN8*, acting as a positive regulator (Miller et al., 2008). Thus, *ZmGI* might repress flowering in LD condition *via* a *ZmGI*-*ZmCONZ1*-*ZmZCN8* regulatory module.

In maize, an important flowering time QTL has been identified, *VEGETATIVE TO GENERATIVE TRANSITION 1* (Vlăduţu et al., 1999). *VGT1* corresponds to a noncoding regulator of the *AP2*-like TF, ZmRAP2.7. *ZmRAP2.7* functions as a negative regulator of maize flowering (Salvi et al., 2007). A MADS-box TF, *ZmMADS69* was identified as the causal gene at the *VGT3* QTL, functioning as a constitutive activator of flowering (Castelletti et al., 2020). Maize also contains the *ZmMADS69*-*ZmRAP2.7*-*ZmZCN8* regulatory module that functions in both LD and SD conditions. *ZmMADS69* functions as a flowering activator by suppressing expression of the flowering repressor *ZmRAP2.7*, thereby relieving its transcriptional repression of the florigen gene *ZmZCN8* to induce early flowering (Liang et al., 2019). *ZmZCN8* is transcribed and translated in the leaf vasculature, then moves through the phloem to the SAM where it interacts with DELAYED FLOWERING 1 (DLF1), a homolog of the Arabidopsis FD protein, to activate downstream floral organ identity genes like *ZEA MAYS MADS-BOX 4* (*ZmZMM4*) (Muszynski et al., 2006; Danilevskaya et al., 2008; Lazakis et al., 2011; Meng et al., 2011).

## 4 Genotypic variation helps crops adapt to different latitudes

When LD and SD crops move out of their native ranges and adapt to new locations, they must alter their flowering behavior and become less sensitive to photoperiods to ensure reproductive success in their non-native zones. During this process, natural and artificial selection act on genotypic variation to produce individuals that harbor suitable alleles and flower optimally, becoming locally adapted.

In the LD crops barley and wheat, major regulators conferring photoperiod sensitivity are encoded by *PPD1* genes. *PPD1* was identified as the core determinant of photoperiod responses in barley (Turner et al., 2005). The recessive *ppd1* allele was selected for in barley from northern Europe, conferring delayed flowering and maturity in LD conditions. The dominant allele *PPD1*, however, was selected for in barley from southern Europe, promoting flowering in response to longer days (Turner et al., 2005; Hemming et al., 2008; Jones et al., 2008). Similar to barley, photoperiod-sensitive wheat is stimulated to flower only after exposure to long days, and flowering is delayed during short days. Photoperiod-insensitive wheat flowers independently of day length and can be grown at lower latitudes (Worland and Snape, 2001). In wheat, dominant *PPD1* greatly reduce sensitivity to photoperiod and confer early flowering phenotype under both LD and SD conditions, resulting yield benefits in Europe (Cockram et al., 2007). Another gene involved in adaptation of LD crops to different latitudes is *PPD2*. In barley, the dominant *PPD2* allele was selected for in spring cultivars at low latitudes to promote flowering in SD conditions, while the recessive *ppd2* allele was selected for in winter cultivars grown at higher latitudes (Halliwell et al., 2016).

Wild peas display a typical winter habit, which consists of germination in autumn, vegetative growth during winter, and flowering in response to long days in spring (Abbo et al., 2003). However, the majority of cultivated peas can flower in SD and are grown as a spring crop, suggesting this ability has been an important factor for the expansion of pea cultivation (Lejeune-Hénaut et al., 1999). Four flowering loci *HR*, *SN*, *LATE FLOWERING* (*LF*), and *EARLY* (*E*) have been found to contribute to this variation (Foucher et al., 2003; Weller et al., 2012; Liew et al., 2014). The recessive *hr* allele causes early flowering in SD and decreases photoperiod response (Weller et al., 2012). *sn* mutants flowered early in SD conditions and eliminated PS (Liew et al., 2014). *lf* mutants displayed an extremely early, photoperiod-insensitive initiation of flowering (Foucher et al., 2003). The *E* locus can promote flowering without influencing the general PS of the plant, but the mechanism is not well understood at the molecular level (Lejeune-Hénaut et al., 2008; Weller et al., 2012). Various allelic combinations of *HR*, *SN*, *LF*, and *E* confer a wide range of flowering times in various conditions. The *lf sn* allelic combination, for instance, contributes to extremely early flowering and complete photoperiod insensitivity. The *LF SN HR e* allelic combination, however, contributes to late flowering in LD conditions and completely prevents flowering in SD conditions (Murfet, 1985; Weller et al., 2012). Most spring flowering (*hr*) pea cultivars carry at least one additional mutation of *sn* or *lf* alleles, with many also carrying mutations at the *E* locus (Weller and Ortega, 2015).

In rice, *Hd1*, *Ghd7*, *DTH8*, and *OsPRR37* are core genes regulating flowering, and different combinations of these genes determine the photoperiod response and latitudinal adaptability of rice (Zhang et al., 2015; Zhang et al., 2019; Zong et al., 2021). The wild rice *O. rufipogon*, which is grown in tropical and subtropical regions of Asia, has strong PS and contains functional *Hd1*, *Ghd7*, *DTH8*, and *OsPRR37* alleles. As wild rice evolved into modern varieties with different levels of PS, various allelic combinations of these genes were selected for to adapt rice to different latitudes. The sixteen possible allelic combinations of these four genes can be divided into three main groups with different PS. The first group exhibits strong PS and contains either four functional alleles (HGDP), three functional alleles (HGDp, HgDP, HGdP, hGDP), or functional alleles of only *Hd1* and *Ghd7* (HGdp). These combinations lead to a long vegetative growth phase and plants carrying them are mainly cultivated in tropical and subtropical regions of China. Rice cultivars containing combination of *Hd1*, *Ghd7* and *Hd1, Ghd7*, *DTH8* have LD repression and SD promotion effects, resulting sufficient vegetative growth for maxima photoassimilation and higher yield under LD conditions (Sun et al., 2022). The second group exhibits no PS and contains only a functional *Hd1* allele (Hgdp) or four non-functional alleles (hgdp). These combinations lead to early heading dates and are generally cultivated in the northern part of China. The third group exhibits moderate photoperiod sensitivity contains the other eight allelic combinations, and is suitable for planting in the middle latitudes of China (Zhang et al., 2015; Zong et al., 2021; Chen et al., 2022). In summary, non-functional alleles of *Hd1*, *Ghd7*, *DTH8*, and *OsPRR37* allow rice to be grown at higher latitudes, while functional alleles facilitate adaptation to lower latitudes. In addition, variation in other flowering-related genes also helps rice to adapt to different latitudes. For example, as rice began to be cultivated at higher latitudes, a functional early-heading *RFT1* allele was selected for, while the late-heading non-functional *rft1* allele was retained in wild or cultivated rice grown at lower latitudes (Ogiso-Tanaka et al., 2013; Naranjo et al., 2014; Zhao et al., 2015). In rice breading, the late-heading allele could be utilized for increasing yield when growth duration is not limited. The early-heading allele is preferred when the constraint comes in multiple season-cropping systems and in the northernmost region of rice cultivation (Zhu et al., 2017). Non-functional alleles of *Hd6* and *Hd16* also contributed to the expansion of rice cultivation to higher latitudes (Kwon et al., 2014; Nemoto et al., 2018).

Soybean, as a SD crop, became acclimated to LD conditions at higher latitudes in Asia and North America by accumulating early-flowering alleles to reduce or completely eliminate its photoperiod sensitivity. *Tof11* and *Tof12* have played essential roles in soybean domestication for growth at high latitudes. The *tof12* mutation has been selected for in cultivated soybean, resulting in earlier flowering and maturity. The *tof11* mutation, which occurred after that of *tof12*, further accelerated flowering and maturity, also contributing to adaptation to higher latitudes (Li et al., 2019; Li et al., 2020; Lu et al., 2020). As plants acclimated to higher latitudes, different combinations of *E1*/*E1lb*, *E3*, and *E4* alleles were selected for, such as *e3e4*, *e1e3e4*, *e1e3*, *e1e4*, *e1-ase3* and *e1-ase1lbe3* (Xu et al., 2013; Zhu et al., 2019). *e2*, which leads to an early flowering phenotype, is prevalent in soybean cultivated in northern China (Langewisch et al., 2014; Wang et al., 2016). Recent research found that the *Tof5^H1^* allele was artificially selected for in cultivated soybean and promotes adaptation to higher latitudes. Moreover, the early flowering allele *Tof18^G^* promotes adaptation to high latitudes in both cultivated and wild soybean (Kou et al., 2022).

Conversely, for soybean to acclimate to the SD conditions at lower latitudes in Brazil, required a long juvenile (LJ) trait to delay flowering time and improve yield. When soybean was first imported to Brazil from North America, it could only be cultivated farther south than 22°S. This barrier remained until the LJ trait was identified and introduced into soybean cultivars in central-western Brazil in 1970, allowing soybean production to expand to lower latitude regions and even to the equator (Lin et al., 2021b). *J* is the main locus regulating the LJ trait. Loss-of-function mutations in *J* can increase soybean yield by 30–50% by prolonging the flowering phase (Lu et al., 2017; Fang et al., 2021). A recent study identified a novel locus, *Tof16*, that delays flowering time and improves yield at low latitudes. Mutations in *Tof16* and *J* were gradually selected for as soybean acclimated to tropical regions. When soybean was initially disseminated to lower latitudes, weak *tof16* and *j* mutants were selected for, leading to delayed flowering. However, these weak mutations in *tof16* and *j* did not lead to complete adaptation to tropical regions. Thus, null alleles of *tof16* and *j* were selected for based on the two earlier weak alleles, which prolonged the flowering period even further and improved soybean yield in tropical regions (Dong et al., 2021). In addition, the *ft2aft5a* double mutant could overcome the genetic compensation effect and showed an enhanced LJ phenotype and high yield at low latitudes (Li et al., 2021). Moreover, the *lux1lux2* double mutant of soybean completely lost photoperiod sensitivity, resulting in extremely late flowering. This phenotype was similar to the famous photoperiod insensitive tobacco (*Nicotiana tabacum*) mutant Maryland Mammoth. Thus, *lux1lux2* was named the Guangzhou Mammoth (Bu et al., 2021). All these alleles and varieties provided important genetic resources for improving soybean yield in tropical areas.

In maize, many genes controlling flowering time have been found to play important roles in the expansion of cultivation from tropical and subtropical regions to higher latitudes. *ZmCOL3* is a repressor of flowering that functions under LD conditions. The loss of one cytosine in the 3’UTR of *ZmCOL3* and the presence of a 551bp fragment in the promoter region have been found to reduce transcription of *ZmCOL3* and help maize adapt to temperate regions (Jin et al., 2018). Temperate maize exhibits higher *ZmMADS69* expression than tropical maize in both the apex and leaf tip tissues, which indicates *ZmMADS69* might have been selected for as maize adapted to temperate regions (Liang et al., 2019). Defective alleles of *ZmCCT9* and *ZmCCT10* were selected for in maize cultivars that are grown in North and South America; these alleles result in the activation of florigen *ZmZCN8* and consequently accelerated flowering in LD conditions (Yang et al., 2013; Guo et al., 2018; Huang et al., 2018). A miniature transposon (MITE) inserted 70kb upstream of *ZmRAP2.7* was another major target of selection and contributed to adaptation of maize to temperate regions (Ducrocq et al., 2008). Two genotypes, SNP-1245A and Indel-2339 in the promoter of *ZmZCN8*, have also been identified. The early flowering SNP-1245A allele was initially selected for during the early domestication of maize. The Indel-2339 allele was later introgressed into the SNP-1235A haplotype and was subsequently selected for as maize cultivation expanded from its tropical origin to more temperate regions (Guo et al., 2018). In summary, the *ZmMADS69*-*ZmRAP2.7*-*ZmZCN8* regulatory module has been targeted by selection and contributed to the expansion of maize cultivation to higher latitudes.

## 5 Future perspectives

**Figure 4:**
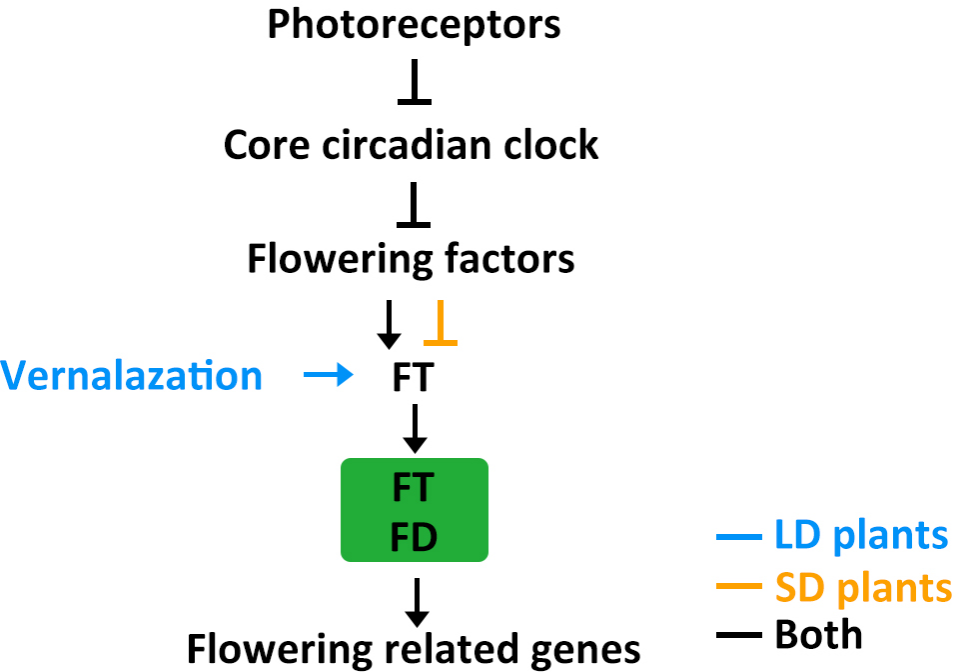
The putative model of flowering regulatory module in LD crops and SD crops. Positive and negative regulatory actions are indicated by arrows and lines with bares, respectively.

In this review, we discussed recent studies in the field of flowering time regulation in LD crops like wheat, barley, and pea as well as SD crops like rice, soybean, and maize. In these major crops, flowering time is regulated by genetic networks that respond to day length. All LD crops and SD crops have similar regulatory modules that control flowering time (**Figure 4**).

Photoreceptors detect light signals and transmit this information to the circadian clock through several different signalling mechanisms. The circadian clock then integrates the light cues and regulates flowering factors. Flowering factors modulate expression of the florigen gene *FT*. FT moves from leaves to the SAM and interacts with FD to activate flowering related genes, such as *AP1* and *FUL*. The core circadian clock genes *ELF3*, *ELF4*, *LUX*, and *PRRs* are conserved in crops and play important roles in natural adaptation to different latitudes. The FT-FD complex is also conserved in crops and further regulates downstream flowering related genes, such as *AP1*/*FUL*-like genes like *VRN1* in wheat, *VEG1*, *PIM*, and *UNI* in pea, *OsMADS14*, *OsMADS15*, and *OsMADS18* in rice, *GmAP1* in soybean, and *ZmZMM4* in maize (Hofer et al., 1997; Taylor et al., 2002; Danilevskaya et al., 2008; Li and Dubcovsky, 2008; Berbel et al., 2012; Kobayashi et al., 2012; Nan et al., 2014; Yin et al., 2019; Dong et al., 2022). Usually, the flowering factor is a homolog of Arabidopsis *CO*, such as *CO* in wheat and barley, *Hd1* in rice, and *CONZ1* in maize (Griffiths et al., 2003; Miller et al., 2008). However, in addition to the conserved *CO-FT* module, rice has specifically evolved the *(Hd1/Ghd7/DTH8)-Ehd1-Hd3a/RFT1* pathway (Zong et al., 2021). The interplay between SD-promotion and LD-repression pathways determines the differential effects of daylength on rice heading, highlighting the genetic diversity of flowering control mechanisms in crops. Soybean, in addition to having Arabidopsis *CO* homologs, also possesses the unique central flowering factor *E1*. Further research is needed, however, to fully understand why this specific central flowering factor evolved in soybean (Xu et al., 2015). For example, it is unknown whether *E1* also appeared in other crops but disappeared during evolutionary history or was selected against during domestication and improvement.

Both LD and SD crops require changes in their flowering behavior when they move away from their native ranges and adapt to a new locality. In general, they become less sensitive to photoperiods to ensure reproductive success in non-native zones. However, different crops have evolved different strategies for adjusting their flowering time. For example, crops differ in the role played by other agronomic traits, such as temperature tolerance, during adaptation. Natural variation in flowering time regulation in SD crops often involves just photoperiod-related genes, but in many LD crops it may also involve low temperature-related genes (**Figure 4**) (Lin et al., 2021a). These differences may reflect where these lineages evolved: SD crops evolved in equatorial regions characterized by stable temperatures and daylengths throughout the year, while LD crops evolved in temperate regions with fluctuating temperatures and changing daylengths. SD crops mainly developed photoperiodic control of flowering, while LD crops acquired additional vernalization requirements as an adaptation to the cold. Further studies are needed to systematically evaluate natural variation in other agronomic traits that influence flowering time. This information would help clarify the evolution of flowering regulatory pathways and possibly help generate new cultivars with improved yields.

As major crops were disseminated to new continents, landraces were developed by farmers as the outcome of artificial selection that facilitated adaptation to the new environments. Subsequently, breeders used introduction, selection, and cross breeding to introduce desirable agronomic traits into crops and generate commercial cultivars. Different allelic combinations, such as combinations of *Hd1*, *Ghd7*, *DTH8*, and *OsPRR37* in rice, could help crops withstand a range of ecological and climatic conditions (Zhang et al., 2015; Zong et al., 2021; Chen et al., 2022). Thus, by investigating the great genetic diversity in crop populations, reintroducing useful genetic resources into crops, and exploiting appropriate gene combinations during breeding, farmers and breeders may be able to improve yields and increase the agricultural and geographic flexibility of crops in the future.

In summary, a comprehensive understanding of the molecular networks regulating flowering time in different crops is needed to maximize production. Additionally, it is important for breeders to investigate variation in flowering time regulation, including how key genes are retained or lost through the process of crop domestication, in order to reveal the crops’ histories, select better alleles, and develop improved cultivars for future breeding applications.

## Author contributions

FW drafted the manuscript. SLi and FK revised the manuscript. XL and SLu supervised this work and revised the manuscript. All authors contributed to the article and approved the submitted version.
